# Preliminary results for salt aerosol production intended for marine cloud brightening, using effervescent spray atomization

**DOI:** 10.1098/rsta.2014.0055

**Published:** 2014-12-28

**Authors:** Gary Cooper, Jack Foster, Lee Galbraith, Sudhanshu Jain, Armand Neukermans, Bob Ormond

**Affiliations:** 1225 East Arques Avenue, Sunnyvale, CA 94085, USA

**Keywords:** salt aerosol production, marine cloud brightening, effervescent spray atomization

## Abstract

The large-scale production of vast numbers of suitable salt nuclei and their upward launch is one of the main technological barriers to the experimental testing of marine cloud brightening (MCB). Very promising, though not definitive, results have been obtained using an adapted version of effervescent spray atomization. The process is simple, robust and inexpensive. This form of effervescent spraying uses only pressurized water and air sprayed from small nozzles to obtain very fine distributions. While it is far from optimized, and may not be the best method if full deployment is ever desired, we believe that even in its present form the process would lend itself well to preliminary field test investigations of MCB. Measurements obtained using standard aerosol instrumentation show approximately lognormal distributions of salt nuclei with median diameters of approximately 65 nm and geometric standard deviations slightly less than 2. However, these measurements are not in agreement with those based on scanning electron microscopy imaging of collected particles, an observation that has not yet been explained. Assuming the above distribution, 10^15^ particles per second could be made with 21 kW of spray power, using approximately 200 nozzles. It is envisioned that existing snow making equipment can be adapted to launch the nuclei 60–100 m into the air, requiring approximately 20 kW of additional power.

## 1. Introduction

Marine cloud brightening (MCB), one of several solar radiation management geoengineering ideas involving the production of global cooling aerosols to compensate for the warming associated with continuing fossil fuel burning, was first postulated by Latham [1,2]. The basic principle behind the idea is to seed marine stratocumulus clouds with seawater aerosol generated at or near the ocean surface. Most particles would have sufficiently large salt mass to ensure their activation and subsequent growth within the clouds, without being so large as to encourage precipitation. Moreover, they would be sufficiently numerous so as to enhance the cloud droplet number concentration to values substantially higher than the natural level, thereby enhancing the cloud albedo [3,4].

Major advantages of using MCB as compared to other geoengineering proposals, such as injecting sulfur into the stratosphere (which can deplete stratospheric ozone), include the ability to localize application, the relatively rapid reduction of most effects on cessation of spraying, and the fact that seawater is non-polluting and non-toxic. As with all proposed geoengineering methods, the possibility of unintended consequences cannot be excluded.

We have been studying various techniques for salt spray production for some time and our earlier results have been reported [5–7]. Briefly reviewing, the most obvious way to obtain monodisperse droplets is to spray through sub-micrometre orifices in the Rayleigh mode [8]. Our efforts at keeping 0.5 μm orifices open over extended periods were unsuccessful [6], even using continuous filtering of the liquid. The amount of seawater that would need to be sprayed to produce 10^17^ nuclei s^−1^ through 0.4 μm holes, as proposed by Salter *et al*. [8], would be of the order of 30 l s^−1^. Spraying this amount of liquid from a concentrated source is bound to give rise to substantial evaporative cooling of the surrounding air, which could, because of the stratification thus created, impede the rise of the nuclei into the clouds.

For these reasons, we have concentrated on spraying minimum quantities of water, while preserving the required number and minimum size of the resulting nuclei. Another method we have studied is the formation of electrically driven Taylor cone jets [6] from saltwater, which fortuitously produces relatively narrow size distributions of salt particles with median distribution diameters of 60–83 nm. However, in order to produce 10^17^ particles s^−1^ this method would require the formation of some 10^8^ Taylor cones. The creation of a spraying structure with such a number of emitters would be expensive and an undertaking that is obviously not trivial. The enormous electrical space charge created by these very highly charged particles creates additional handling problems.

A recently described method [7] involves the spraying of saltwater at or near its critical point. As water is heated at its critical pressure, its surface tension continuously decreases and eventually becomes zero at the critical point (about 374°C and 22.1 MPa), at which point it becomes a supercritical fluid. The behaviour of water in this form is almost like that of a gas, although one of high density. Salt water under supercritical conditions also sprays like a gas and produces sub-micrometre salt particles. Our efforts produced lognormal particle-size distributions having median diameters of 32–286 nm, with geometric standard deviations (GSDs) around 2. The energy required for implementation would, however, be excessive, and the corrosion problems associated with supercritical saltwater are substantial.

We present here preliminary results for the latest method we have investigated, effervescent spray, the main advantages of which are simplicity, low cost and robustness. A brief discussion of the background and theory of effervescent spray, a description of our experimental apparatus, and results of experiments using the apparatus for the production of salt aerosol of the size range and particle flux required for effective utilization of MCB are included.

## 2. Effervescent spray

Effervescent spray atomization (ESA) is achieved by combining a liquid and a pressurized gas before releasing the mixture through a nozzle. The resulting rapid expansion of the gas at the nozzle exit causes enhanced breakup of the liquid, and results in significantly enhanced atomization. Chawla [9,10] attributes atomization of two-phase fluid spray to choked flow and the lower speed of sound for the mixture. Choked flow results in a substantial pressure jump just outside the orifice thereby shattering drops explosively. Sovani *et al*. [11] reviewed effervescent atomization in great detail and summarized the results of 64 investigators. Most applications produce droplets in the tens of micrometre range and use gas-to-liquid mass ratios (GLRs) of a few per cent. Typically, a large exit orifice (millimetre-sized) can be used, with large flows, and hence clogging is of little concern. In many applications, initial droplets are small at the nozzle exit, but may grow by a factor of three to four due to coalescence along the spray axis [12,13].

Effervescent spray has been suggested as a possible candidate for MCB salt spray by Guildenbecher *et al*. [14], following a discussion with some of the present authors. Experiments with effervescent diesel fuel atomizers at Purdue University [15] produced droplet distributions with Sauter mean diameters (SMDs) of 2.5 μm at GLRs of 1.5–4% (SMD is defined as the diameter of a drop that has the same volume-to-surface ratio as the sum of the entire collection of drops).

Caputo *et al*. [16,17] reported production of 1 μm SMD water droplets using two-phase flow of water and carbon dioxide gas at GLRs of 4–5, with surprisingly narrow size distributions in some cases. Using nitrogen as the mixing gas with water, an SMD of 1 μm was obtained at a GLR of 1 at around 8.5 MPa. Note that since the vast majority of the required power is associated with compression of the gas, the use of a low GLR is highly desirable. We report here on some experiments using pressures around 8 MPa, and GLRs between 0.1 and 1.

It is instructive to relate the SMD of the droplet distribution to its mode diameter, i.e. the peak in the distribution curve or the diameter most frequently observed. Since our concern is with the number of suitable nuclei produced, this parameter is most relevant. Most of the spray distributions are lognormal in shape, with a GSD of approximately 2. Under these conditions, using the Hatch–Choate equations [18], it is found that D_32_=5.35 times the mode diameter of the droplet distribution. For a 3.2% salt solution, there is a further fourfold reduction in diameter upon evaporation. Hence the mode diameter of the dry salt crystals is approximately 21 times smaller than the D_32_ of the droplets. Hence an SMD of 2 μm should produce a peak count around 100 nm for the salt nuclei, well within the desired range [19].

## 3. Description of experimental apparatus

**Figure 1.**
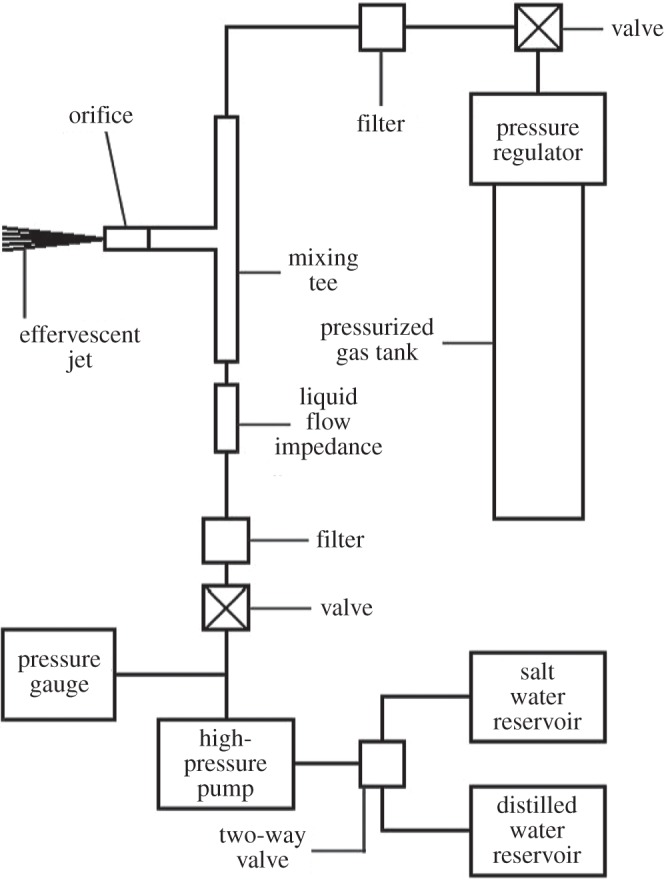
Schematic of the effervescent spray apparatus for producing nanometre-sized NaCl particles.

A schematic of the apparatus for effervescent spray of salt water through a small orifice is shown in figure 1. It comprises a pump with two liquid reservoirs, one containing saltwater and the other containing distilled water for flushing, both connected alternately with a two-way valve to the pump inlet. The Haskel model ASF-60 pump uses compressed air (not shown) on a large piston to drive a small piston that pumps the liquid, giving a pressure boost of 69 : 1. The pump's fluid cylinder has a capacity of 11 ml. Given the known stroke volume, the time interval between strokes provides a reliable measurement of the liquid flow rate. Following the pump is a pressure gauge, an on–off valve and a filter. The fluid flows through a capillary tubing flow impedance into an adapted Valco HPLC post-column reaction tee where mixing occurs and thence to the spray nozzle. A gas supply, connected to the other input of the mixing tee, consists of a compressed gas cylinder, a pressure regulator, an on–off valve, a filter and a check valve.

Rather than a separate mixing chamber, as is found in most effervescent spray apparatus, the device relies on turbulent mixing in the thin tubes of the tee. The diamond, sapphire or stainless steel nozzle orifices range from 50 to 200 μm in diameter. Pressures typically range from 8 to 9 MPa.

**Figure 2.**
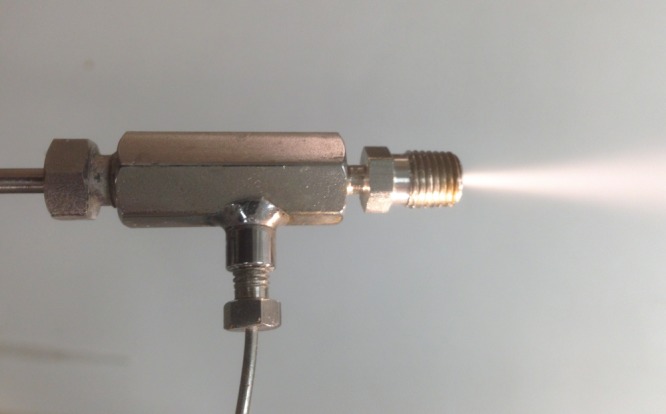
Effervescent nozzle with gas entering from the left, liquid entering from the lower flow impedance tubing and the sapphire orifice imbedded in the stainless steel nozzle on the right. (Online version in colour.)

Upon addition of gas at the appropriate pressure, the initially fine liquid jet is observed to explosively diverge from the orifice into an approximately 2 mm wide, 90° cone at the nozzle exit before projecting forward (figure 2).

To determine the GLR, the mass flow of gas and liquid needs to be measured simultaneously while in operation. The liquid flow is determined from the pump stroke timing, whereas the gas is gravitationally separated from the fluid and the resulting wet gas flow is determined by measuring the pressure drop in a tube of known length and diameter. We are developing a semi-empirical mathematical model for the mixed flow that gives reasonable predictions of the observed results.

An effervescent jet assembled with a 125 μm diameter sapphire nozzle and commercial tube fittings is shown in figure 2.

The spray was produced in a 7.3×7.9×2.7 m room with a total volume of 156 m^3^ where the droplets evaporated, leaving primarily NaCl crystals. A single nozzle could produce detectable room clouding in a matter of minutes. A large centrifugal fan continually mixed and circulated room air and salt aerosol. The room humidity was kept below 40% RH, including the short (2–4 min) spray interval.

**Figure 3.**
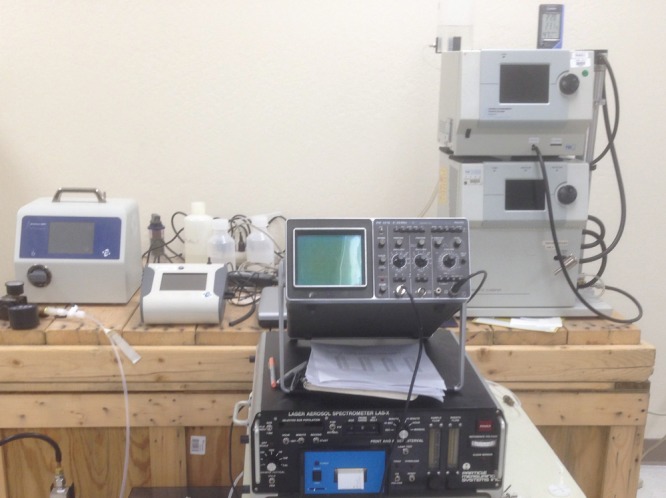
Aerosol measuring instruments used in this work. From right to left are the TSI 3080/3081 classifier/DMA with 3776 CPC on top, the PMS LAS-X optical analyser, the TSI 3330 optical particle sizer and the TSI 3910 NanoScan SMPS. (Online version in colour.)

The resulting aerosol was analysed using the following commercial particle sizing instruments, shown right to left in figure 3:

— a TSI model 3080 classifier/3081 DMA (differential mobility analyser) with a model 3776 CPC (condensation particle counter), a standard instrument combination for sizing aerosols in the 20–700 nm range using electrical mobility analysis, and commonly referred to as an SMPS (scanning mobility particle sizer spectrometer);
— a Particle Measuring Systems (PMS) model LAS-X 1000 optical analyser for sizing aerosol in the tail end of the particle distribution (0.12–3 μm);
— a TSI model 3330 OPS (optical particle sizer) with a range of 0.3–10 μm; and
— a TSI model 3910 NanoScan SMPS with a range 10–420 nm, likewise an electrical mobility analyser.

And, as in previous work, aerosol was deposited on silicon wafers using a flow-through electrostatic precipitator, and the deposit analysed by scanning electron microscopy (SEM) with digital image analysis using the ImageJ software package developed at the US National Institutes of Health.

The interpretation of the resulting distributions proved to be very challenging. To verify the correct operation of the TSI 3080/3081/3776 SMPS, monodisperse latex spheres of known size, dispersed by a TSI model 3076 aerosol generator, were analysed and found to be correctly sized. The same spheres, selected by the 3081 DMA, were approximately correctly sized by the PMS LAS-X optical analyser. Likewise, sprayed salt particles, selected by the 3081 DMA to be, for example, 300 nm, were approximately correctly sized.

There was some concern with aerosol charge. In principle, this atomization method, producing very thin cylindrical sheets of fluid moving at near sonic speed over a nozzle surface, might produce a highly charged aerosol through the triboelectric effect. A spray current of 1.2 μA was measured for a 0.41 g s^−1^ mass flow, with positive particle charge, but this was deemed insufficient to produce Coulombic charged droplet disintegration. Numerous highly charged particles might, however, overwhelm the Kr-85 neutralizer in the TSI 3080 classifier and thereby be interpreted as particles of smaller diameter in the DMA. When a 20 mCi polonium neutralizer was added to the sample inlet no observable size distribution changes were noted, confirming that the Kr-85 neutralizer was able to produce the equilibrium distribution of particle charges that the instrument needs in order to accurately size the particles using electrical mobility measurement.

For all of these reasons, the TSI 3080/3081/3776 SMPS was accepted as the most reliable of the measurement systems. However, these measurements have yet to be brought into agreement with SEM measurements obtained from two separate instruments (as will be discussed).

## 4. Experimental results

**Figure 4.**
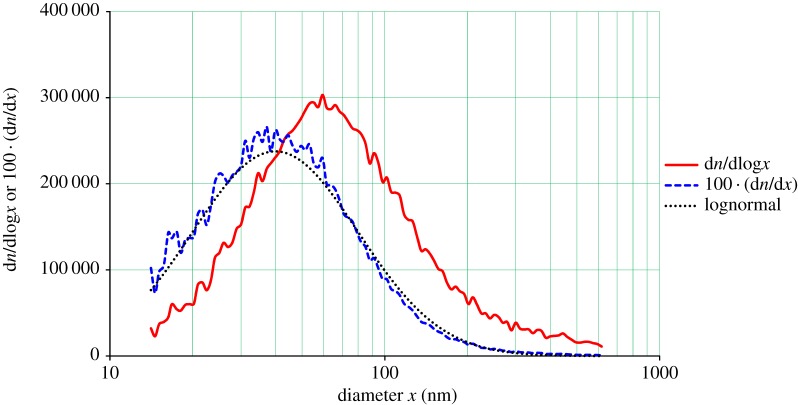
Typical frequency versus diameter plots from the TSI 3080/3081/3776 SMPS for aerosol produced by spraying 3.2% NaCl solution. (Online version in colour.)

Figure 4 shows a typical size distribution of particles as measured with the TSI 3080/3081/3776 SMPS for a 3.2% NaCl solution sprayed at 8.7 MPa gas pressure. The measurement is taken during a 2 min sampling period after the end of the spray period. The solid red curve is the d*n*/dlog*x* observed instrument data, the dashed blue curve is the same data plotted as d*n*/d*x*, which is seen to be in remarkably good agreement with a lognormal distribution (dotted black curve) having a CMD (count median diameter) of 63 nm and a GSD of 2.0, with the peak count occuring at 40 nm. This size distribution and numerous others like it observed under various spray conditions is substantially smaller than would be expected based on the current theories of effervescent spraying. This could be due to additional droplet disintegration caused by dissolved gas expansion, but since the mixing region is very short, gas dissolution should not be significant. Most if not all past ESA measurements were, however, concerned with the sprayed droplet distribution, not that of a solid remaining after evaporation. Based on this distribution (i.e. neglecting momentarily the very significant long tail at larger sizes, which is not lognormal), each nozzle would deliver 5.3×10^12^ particles s^−1^. Thus, one would need only 187 nozzles to produce 10^15^ particles s^−1^, proposed to be a rate useful for preliminary field experiments [5], with a spray rate of 0.08 l s^−1^ and requiring 21 kW of power.

Given the latest thinking on the salt nucleus size distribution desired for MCB [19], our 63 nm distribution, while not ideal, is definitely considered useful for preliminary experimentation in MCB. This size distribution is, however, not in agreement with SEM measurements, being consistently lower by a factor of 2–3 in the location of the mode diameter. SEM measurements, while more direct than electrical mobility measurements, have sampling and image analysis problems that can significantly skew the distribution.

To try to resolve the apparent problem, measurements were made with all instruments simultaneously. In this test, a 3.2% NaCl solution was sprayed with a 126 μm diameter nozzle at 8.7 MPa gas pressure and 9.44 MPa liquid pressure using a 305 μm×25 cm liquid flow impedance, with a nozzle input pressure calculated to be 6.32 MPa. Liquid flow was 0.41 ml s^−1^ and gas flow was 0.122 l s^−1^ for a GLR of 0.345.

**Figure 5.**
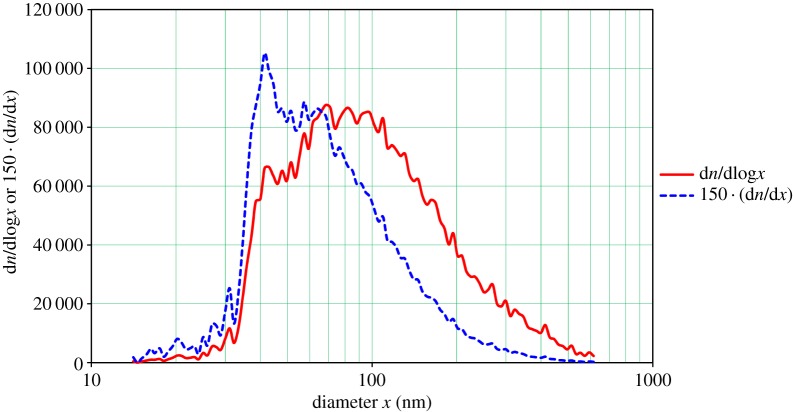
Frequency versus diameter plots from the TSI 3080/3081/3776 SMPS for aerosol produced by spraying 3.2% NaCl solution (Ref: 4/3/2014; 15:37:30 to 15:41:30). (Online version in colour.)

The TSI 3080/3081/3776 SMPS results are illustrated in figure 5 for both d*n*/dlog*x* (solid red curve) and d*n*/d*x* (dashed blue curve). The CMD of the distribution is found to be 89.6 nm with a GSD of 1.87. Again, neglecting the long tail of the distribution, this arrangement would deliver 2.8×10^12^ particles s^−1^, requiring 350 nozzles, a total power of some 40 kW and a spray of 0.14 l s^−1^ to achieve the target particle production rate of 10^15^ particles s^−1^.

The TSI 3910 NanoScan instrument, which was found to be seriously challenged to make measurements above 140 nm, gave a distribution with a CMD of 45.2 nm and a GSD of 1.83.

**Figure 6.**
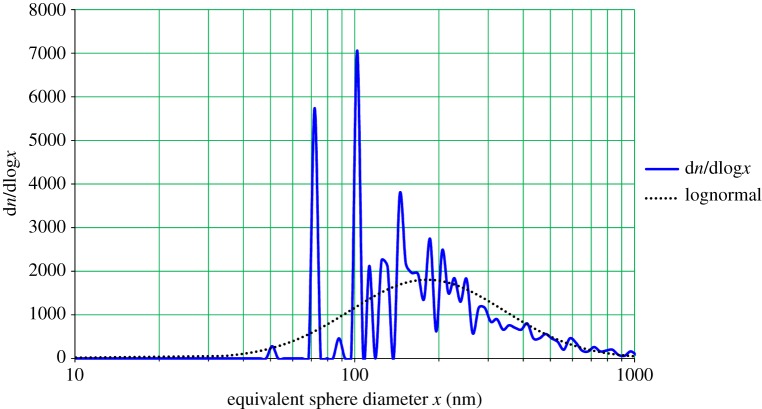
Frequency versus diameter plots from the Hitachi 4010 SEM for aerosol produced by spraying 3.2% NaCl solution with solute precipitation on a silicon wafer in an electrostatic precipitator (Ref: 4/3/2014; 15:41). (Online version in colour.)

Figure 6 shows the particle-size distribution determined by analysis of SEM images of salt particles collected on a silicon wafer during real-time analysis by the various particle sizing instruments. The resulting lognormally fitted distribution is seen to have a CMD of 181 nm and a GSD of 1.89. While the GSD is similar, the geometric mean is almost twice the size of the one observed with the SMPS. The discontinuous nature of the curve at small diameters is an artefact of the digitization of the image where, for example, a single two-dimensional pixel represents particles with an equivalent (three-dimensional) sphere diameter of 50 nm.

**Figure 7.**
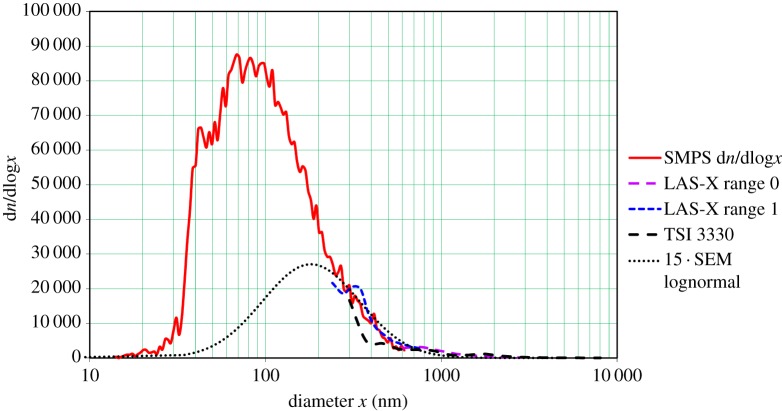
Frequency versus diameter plots from the TSI 3330 OPS and LAS-X scaled and added to those obtained from the TSI 3080/3081/3776 SMPS and from SEM measurements, for aerosol produced by spraying 3.2% NaCl solution (Ref: 4/3/2014; 15:37:30 to 15:41:30). (Online version in colour.)

This might imply that the distribution could have a peak in the region above 700 nm, where the SMPS cannot measure. To this end, the large end of diminutions as measured by both optical instruments with respective ranges of 0.12–3 μm and 0.3–10 μm were fitted to the distribution measured by the SMPS, and the lognormal curve from the SEM measurement was added. The combined result, shown in figure 7, confirms that the curve is generally a smooth descending curve, without secondary peaks from the larger particles.

## 5. Discussion of the results

The disagreement between the SMPS and SEM results is substantial and striking. While there may be some image digitization effects in the SEM measurement algorithms, resulting in the spiky behaviour at low sizes, visual inspection shows, over many pictures, that the background is almost entirely devoid of any particles in the 40 nm region. Both 20 kV and 5 kV instruments were used in these measurements. Since the SEM inspects only a very small fraction of the wafer (even with multiple observation points) sampling is a potential problem. In the case of supercritical saltwater spray aerosols, even smaller particles were readily identified by SEM [7].

We used the TSI 3080/3081/3776 SMPS to measure the precipitation efficiency of the flow-through electrostatic precipitator by measuring particle concentration versus bin size in its output with the precipitation voltage on and off, using salt aerosol. The results strongly suggest that particles in the 10–40 nm (SMPS) range are precipitated with an efficiency of 96–98%, and those in the 100–700 nm range better than 90%. The missing particles in the SEM are in fact preferentially precipitated, enhancing their chance for being observed.

As mentioned earlier, the SMPS provides only indirect measurements, its mobility results may be influenced by particle shape, preferential orientation, etc., and its results are not always in agreement with other measurements. However, a very thorough and elaborate experimental analysis of salt particles by Zelenyuk *et al*. [20], using a variety of methods, showed that the mobility diameter of single salt particles measured with an SMPS is in good agreement with SEM measurements of these same particles, even for those having multiple charges. This is in agreement with our own, much more limited, observations of single polystyrene latex (PSL) spheres.

To try to resolve the problem, two more instruments were procured: a second SMPS comprising a TSI model 3080 L electrostatic classifier coupled to a TSI model 3025A ultrafine condensation particle counter, and a TSI model 3340 laser aerosol spectrometer, a highly sensitive, high-resolution counter with a measurement range of 0.09–7.5 μm, a significantly improved and updated version of the PMS LAX instrument. Correct operation of both instruments was verified, as before, using monodisperse PSL spheres.

For salt aerosol generated by the effervescent sprayer, the new SMPS instrument routinely measured distributions with a median in the range of 60–80 nm, close to what was observed with the previous SMPS instrument. The TSI 3340 generally showed a monotonically decreasing number of particles versus increasing diameter, but no peak, which is what would be expected for a peak diameter outside the instrument's range. These observations lend credence to the SMPS measurement being correct.

The size distributions of aerosols from sprayed salt solutions of varying concentrations do not follow the cube root dependence on concentrations that would be expected from mass conservation. Spraying a 15% salt solution in our system increased the CMD by a factor of 1.4, not the 1.7 that would be expected from mass conservation. Likewise, for salt solutions, the TSI model 3076 constant output atomizer shows a power-law dependence on concentration with an exponent of 0.14 rather than the expected 0.33, while for dioctyl phthalate, in a similar generator, the 0.33 dependence appears perfectly maintained. This discrepancy has not been resolved.

## 6. Conclusion

While there are critical measurement issues to be resolved, this ESA technique looks extremely promising for MCB applications and for laboratory production of NaCl (or other salt) aerosols for cloud physics investigations. Assuming that the present distribution (figure 5) is as effective for conversion into cloud droplets as estimated [19] and is indeed reasonably representative of the sprayed nuclei distribution, the salient conclusion is that it would require only a few tens of kilowatts of power (virtually all for the air compressor), and a few hundred nozzles spraying a few hundreds of millilitres per second of seawater, to comprise a source delivering 10^15^ nuclei s^−1^. Upward launch of the spray to a height greater than 50 m with adapted snowmaking equipment seems quite feasible.
